# Supplementary material for: Magnitude of standard precautions practices among healthcare workers in health facilities of Low and Middle Income Countries: A systematic review and meta-analysis
Source: PLoS One. 2024 Apr 30;19(4):e0302282. doi: 10.1371/journal.pone.0302282 (PMC11060540; doi:10.1371/journal.pone.0302282)
Supplement: S2 Table — (PDF) [file pone.0302282.s002.pdf]

Study Name	year	year category	Country	Study design	Study setting	Profession	Sampling technique	Types of IPC practices assessed	Event	sample size
Umoh VA et al (2020)	2020	>2015	Nigeria	Cross-sectional	Hospitals all type	All other types of HCWs	Random	All others standard precautions	24	51
Adegboye MB et al (2018)	2018	>2015	Nigeria	Cross-sectional	Hospitals all type	All other types of HCWs	Others	Hand hygiene only	26	80
Mursy SMM et al (2019)	2019	>2015	Sudan	Cross-sectional	Hospitals all type	Nurses only	Others	All others standard precautions	72	110
Desta M et al (2018)	2018	>2015	Ethiopia	Cross-sectional	Hospitals all type	All other types of HCWs	Others	All others standard precautions	86	150
Sarani et al (2015)	2015	<=2015	Iran	Cross-sectional	Hospitals all type	Nurses only	Random	All others standard precautions	71	170
Assefa J et al (2020)	2020	>2015	Ethiopia	Cross-sectional	Hospitals and other healthcare facilities	All other types of HCWs	Random	All others standard precautions	94	171
Iliyasu G et al (2016)	2016	>2015	Nigeria	Cross-sectional	Hospitals all type	All other types of HCWs	Others	Hand hygiene only	130	200
Woldegioris T et al (2019)	2019	>2015	Ethiopia	Cross-sectional	Hospitals all type	Nurses only	Others	All others standard precautions	92	204
Ngwa CH et al (2018)	2018	>2015	Cameron	Cross-sectional	Hospitals all type	All other types of HCWs	Random	All others standard precautions	41	216
Bekele I et al	2018	>2015	Ethiopia	Cross-sectional	Hospitals all type	Nurses only	Others	All others standard precautions	148	231

Suliman M et al (2018)	2018	>2015	Jordan	Cross-sectional	Hospitals all type	Nurses only	Others	All others standard precautions	161	247
Tenna A et al (2013)	2013	<=2015	Ethiopia	Cross-sectional	Hospitals all type	All other types of HCWs	Others	Hand hygiene only	125	261
Askarian M et al (2005)	2005	<=2015	Iran	Cross-sectional	Hospitals all type	Nurses only	Others	All others standard precautions	53	270
Hussen SH et al (2017)	2017	>2015	Ethiopia	Cross-sectional	Hospitals all type	All other types of HCWs	Others	All others standard precautions	164	271
Sahiledengle B (2019)	2019	>2015	Ethiopia	Cross-sectional	Hospitals all type	Nurses only	Random	All others standard precautions	134	273
Yohaness T et al (2019)	2019	>2015	Ethiopia	Cross-sectional	Hospitals all type	All other types of HCWs	Random	All others standard precautions	41	274
Yazie TD et al (2019)	2019	>2015	Ethiopia	Cross-sectional	Hospitals all type	All other types of HCWs	Random	All others standard precautions	162	282
Hang Pham TT et al (2019)	2019	>2015	Vietnam	Cross-sectional	Hospitals and other healthcare facilities	All other types of HCWs	Random	All others standard precautions	151	314
Paudyal P et al (2008)	2008	<=2015	Nepal	Cross-sectional	Hospitals all type	All other types of HCWs	Others	Hand hygiene only	227	324
Temesgen C et al 2014)	2014	<=2015	Ethiopia	Others	Hospitals all type	All other types of HCWs	Others	All others standard precautions	206	326
Engdaw GT et al (2019)	2019	>2015	Ethiopia	Cross-sectional	Hospitals all type	All other types of HCWs	Random	Hand hygiene only	50	335

Gulilat K et al (2014)	2014	<=2015	Ethiopia	Cross-sectional	Hospitals and other healthcare facilities	All other types of HCWs	Random	All others standard precautions	192	354
Ajibola S et al (2014)	2014	<=2015	Nigeria	Cross-sectional	Hospitals all type	All other types of HCWs	Random	All others standard precautions	23	372
Hosseinialhashemi M et al (2015)	2015	<=2015	Iran	Cross-sectional	Hospitals all type	All other types of HCWs	Others	Hand hygiene only	121	377
Mengesha A et al (2020)	2020	>2015	Ethiopia	Cross-sectional	Hospitals all type	Nurses only	Others	All others standard precautions	200	409
Tadesse AW et al (2020)	2020	>2015	Ethiopia	Cross-sectional	Hospitals all type	All other types of HCWs	Random	All others standard precautions	168	422
Gebresillassie A et al (2014)	2014	<=2015	Ethiopia	Cross-sectional	Hospitals and other healthcare facilities	All other types of HCWs	Others	All others standard precautions	207	483
Sahiledengle B et al (2018)	2018	>2015	Ethiopia	Cross-sectional	Hospitals and other healthcare facilities	All other types of HCWs	Others	All others standard precautions	400	605
Arinze-Onyia SU et al (2018)	2018	>2015	Nigeria	Cross-sectional	Hospitals all type	All other types of HCWs	Others	Hand hygiene only	208	629
Geberemariyam BS et al (2018)	2018	>2015	Ethiopia	Cross-sectional	Hospitals and other healthcare facilities	All other types of HCWs	Others	All others standard precautions	235	648

Laraqui O et al (2008)	2008	<=2015	Morocco	Others	Hospitals and other healthcare facilities	All other types of HCWs	Others	All others standard precautions	1368	2086
Kebede B et al (2015)	2015	<=2015	Ethiopia	Cross-sectional	Hospitals all type	All other types of HCWs	Others	Hand hygiene only	13	17
Legese T et al (2015)	2015	<=2015	Ethiopia	Cross-sectional	Hospitals all type	All other types of HCWs	Others	Hand hygiene only	72	73
Alemu B et al (2015)	2015	<=2015	Ethiopia	Cross-sectional	Hospitals all type	All other types of HCWs	Others	Hand hygiene only	32	47
Zewde GT (2019)	2019	>2015	Ethiopia	Cross-sectional	Hospitals all type	All other types of HCWs	Random	Hand hygiene only	93	125
Koech SJ et al 2021	2021	>2015	Keniya	Cross-sectional	Hospitals all type	All other types of HCWs	Random	Hand hygiene only	150	301
Ekwere T et al 2013	2013	<=2015	Nigeria	Cross-sectional	Hospitals all type	All other types of HCWs	Random	Hand hygiene only	301	430
Gajida Au et al 2020	2020	>2015	Nigeria	Cross-sectional	Hospitals all type	All other types of HCWs	Others	Hand hygiene only	236	302
Oluwagbemiga AO et al 2020	2020	>2015	Nigeria	Cross-sectional	Hospitals and other healthcare facilities	All other types of HCWs	Random	Hand hygiene only	70	137
Alemayehu R et al 2016	2016	>2015	Ethiopia	Cross-sectional	Hospitals all type	All other types of HCWs	Random	Hand hygiene only	205	208

Gezie H et al 2019	2019	>2015	Ethiopia	Cross-sectional	Hospitals all type	All other types of HCWs	Random	Hand hygiene only	56	191
Jemal S 2018	2018	>2015	Ethiopia	Cross-sectional	Hospitals all type	All other types of HCWs	Random	Hand hygiene only	39	91
Joshi SK et al 2013	2013	<=2015	Nepal	Cross-sectional	Hospitals all type	All other types of HCWs	Others	Hand hygiene only	302	336
Yallew WW et al 2015	2015	<=2015	Ethiopia	Cross-sectional	Hospitals all type	All other types of HCWs	Others	All others standard precautions	232	422
Asmr Y et al 2019	2019	>2015	Ethiopia	Cross-sectional	Hospitals all type	All other types of HCWs	Others	All others standard precautions	77	128
Abreha N 2018	2018	>2015	Ethiopia	Cross-sectional	Hospitals all type	Nurses only	Others	All others standard precautions	78	108
