# Supplementary material for: Magnitude of standard precautions practices among healthcare workers in health facilities of Low and Middle Income Countries: A systematic review and meta-analysis
Source: PLoS One. 2024 Apr 30;19(4):e0302282. doi: 10.1371/journal.pone.0302282 (PMC11060540; doi:10.1371/journal.pone.0302282)
Supplement: Attachment — Submitted filename: Response to Reviewers.docx [file pone.0302282.s003.docx]

**Authors’ line-by-line response for reviewers**

| **Title** | | **Magnitude of Standard Precautions Practices among Healthcare Workers in Health Facilities of Low and Middle Income Countries: A Systematic Review and Meta-analysis.** | |
| --- | --- | --- | --- |
| **Name of authors** | | Mengistu Yilma, Girma Taye, and Workeabeba Abebe | |
| For Reviewer 1 – First of all, I would like to express my appreciation and gratitude for your valuable comments that significantly improve the quality of our research and for taking the time to review thoroughly. We have tried to make the necessary amendments and additional improvements to the research accordingly. The following are the line-by-line response for your comments | | | |
| No | **Comments** | | **Response** |
| 1. | Abstract: Introduction- the introduction in the abstract should capture the problem and the gap identified in the literature. You may delete some of the general background information already provided with the problem and the gap.  Results: The results should also capture some of the main findings of the study (in accordance with the objectives of this review). | | The comments were well taken and corrected accordingly. We showed the amendment with track change on the manuscript. |
| 2. | Introduction- Please use a box bracket instead to enclose the in text citation. | | We have used Endnote X7 reference manager to cite and it was automatically generated style. |
| 3. | The problem is not well stated. The authors need to further look at the introduction again. The introduction should clearly establish the problem, context and significance. | | The comments were well taken and corrected accordingly. |
| 4 | Line 76:........ The paucity of evidence about the current subject............ this statement is incorrect and only shows you did not do a good review of the literature. the outbreak of COVID-19, Ebola, Flu has drawn more attention to infection prevention practices leading to increased research and publication in this area. In 2023 alone, hundreds of articles have been published various components of infection prevention. | | We made significant amendment on this issue based on the comment. |
| 5 | In the concluding part of your revised introduction, it should capture the implication of your study on healthcare policies. | | Corrected accordingly |
| 6 | Methods  Line 88: Google scholar is not considered as a major database. | | We made clarification on the search process according to the comments. Google scholar was used as additional source. |
| 7 | The authors' approach to searching for articles leaves room for evidence/article selection bias. Additional searches for both published and grey literature should have been done. Mention Google scholar as part of the sites searched for additional literature. | | As you rightly said Google scholar was used as additional resources that contains grey literatures. |
| 8 | Reference list of included articles should have been used as snowball. Hand searches and consultation with experts /personal correspondence should have been mentioned. | | We mentioned this in method part according to the comment |
| 9 | How many reviewers conducted the electronic search for articles and on which day did you begin and when was the final searches completed?  I stand to be corrected but i doubt if you adhered to the reported items outlined in PRISMA.  How were duplicates removed? | | We made clarification on this issue on subtopic “selection and management processes” |
| 10 | Search Strategy  Line 105-128: I doubt if PubMed will understand the search language and give you accurate and precise search outcome. When two words are combined together as infection prevention or infection control, quotation marks are used ("infection prevention") to communicate to PubMed that it is one word. Inclusion | | The search was conducted as you rightly mentioned and the typing errors during write up was corrected according to the comment. |
| 11 | Be specific with the study design type. you mentioned observational studies, were case studies and qualitative research articles included? Please clarify. | | We clarified that according to the comment |
| 12 | The publication year should be indicated in the inclusion : for instance, articles published within the 10 years from 2013 to 2023. | | We included all years and clarified that according to the comment. |
| 13 | Line 150: Its not clear what you mean by poor quality score. line 157: Statement is unclear | | We clarified that based on the comments |
| 14 | Results 16. Line 183-188: Its unthinkable and unrealistic how 112 articles were retrieved from EMBASE, MEDLINE, Global Health, AJOL and Google scholar. I can emphatically say you only searched PubMed and Google scholar. | | We made clarification based on the comments |
| 15 | Page 12: Provide a legend for the table and state the abbreviations in full. | | We corrected it accordingly |
| 16 | Fig 1: PRISMA flow diagram: it is unclear how the authors arrived at the 429 articles from the 8016 remaining articles. | | We clarified this in result section, the subtopic “selection and identification of studies” |
| 17 | Discuss the limitations with the few databases used, purposive inclusion criteria used and the limits placed on language of published articles. | | We addressed this based on the comment |
| For Reviewer 2 – First of all, I would like to express my appreciation and gratitude for your valuable comments that significantly improve the quality of our research and for taking the time to review thoroughly. We have tried to make the necessary amendments and additional improvements to the research accordingly. The following are the line-by-line response for your comments | | | |
| No | **Comments** | | **Response** |
| 1. | Line 54-line 58--I believe these two statements could be put together into a single concise statement. At the moment they appear unwieldy and have a few repetitions. | | The comment was well admitted and corrected accordingly. The corrections are highlighted with track change in the manuscript. |
| 2. | Line 64-line 65--"In recent years, more attention has been given to IPC practices in health care facilities as a result of the occurrence of new and emerging health problems". This is a claim that was not supported. What is the evidence that more attention has been given to IPC practices in recent years? | | This comment was well accepted and corrected |
| 3. | Line 67-line 70--According to the report from the Organization for Economic Co-operation and Development (OECD), it is estimated that promoting simple IPC measures such as hand hygiene could reduce the AMR burden by about 40%. What OECD report is this? The authors need to provide a citation for this report | | We have corrected this based on the comment |
| 4 | Line 72-75--Healthcare associated infections are the major concern worldwide for the health systems. This statement sounds as if this is the only 'major' issue plaguing health care systems. However, this is not necessarily true. What about the rising incidence of non-communicable diseases in LMICs? What about the challenges associated with Universal Health Coverage? It is probably fair to say it is AMONG the issues but not THE MAJOR issue. The same applies to the statement that follows--- "Ebola, MERS, COVID-19, and monkey pox are the major concerns that have imposed an additional burden on the health system in recent years". While these are among the plethora of challenges that affect healthcare systems, they are not the only ones. | | We have made amendment on this issue based on the comments. |
| 5 | In line 75-76, the authors wrote "In addition to the aforementioned situations, the paucity of evidence about the current subject drove the initiation of this meta-analysis"----The problem is not well established here. What shows that there is a paucity of research on this issue? The authors should have teased out the problem in a better way. For example, there may be single studies in LMICs but these are scattered and not pulled together for evidence purposes etc. I feel the problem or question should be framed in such a way that it will inform the need for a systematic review in this area. As it stands, we only have a piecemeal presentation of the main research issue and why it is worth using a systematic review. | | We have made modification and additional improvement on this section based on the comments. |
| 6 | Discussion section--In general, the discussion section is very lame. What do these findings mean in the light of infection control in LMICs? What are the implications for healthcare practice, research and policy? A great number of studies were conducted in Nigeria—how does this impact your interpretation (although the authors talked briefly about Ethiopia)? The authors should use published research to explain and discuss the trends in prevalence of infections and how this should inform the evidence scenario for HAIs in low resource settings moving forward. | | We tried to make improvements on the discussion section by including more comparison based on the available evidences as per the comment. we also include the implications and significance of the study. |
| 7 | There are so many repetitions in the manuscript. For example, under the subsection "Meta-analysis for IPC practices among healthcare workers"--there are so many repetitions there. The authors should check for other statements that are repeated throughout the manuscript. e.g. This systematic review and meta-analysis included 46 papers in total. | | The comment was well taken and corrected. |
| 8 | The article should be edited for grammatical errors. There are so many editorial mistakes in the manuscript. The authors should take some time to read through the document critically and get them corrected. | | The comment was well taken and corrected according to the comments. |
| 9 | The first statement in the discussion section refers to a CDC document. What document is this? A citation is needed there | | It is corrected based on the comment |
| 10 | The conclusion section should present a succinct summary of the findings as well as the key lessons, implications or recommendations regarding how this evidence informs infection control practices and policies for healthcare workers, patients etc | | We modified the conclusion section based on the comment. |
