# Supplementary material for: Magnitude of standard precautions practices among healthcare workers in health facilities of Low and Middle Income Countries: A systematic review and meta-analysis
Source: PLoS One. 2024 Apr 30;19(4):e0302282. doi: 10.1371/journal.pone.0302282 (PMC11060540; doi:10.1371/journal.pone.0302282)
Supplement: Attachment — Submitted filename: Response to Reviewer and editor.docx [file pone.0302282.s004.docx]

**Authors’ line-by-line response for reviewers**

| **Title** | | **Magnitude of Standard Precautions Practices among Healthcare Workers in Health Facilities of Low and Middle Income Countries: A Systematic Review and Meta-analysis.** | |
| --- | --- | --- | --- |
| **Name of authors** | | Mengistu Yilma, Girma Taye, and Workeabeba Abebe | |
| For Reviewer 3 – First of all, I would like to express my appreciation and gratitude for your valuable comments that significantly improve the quality of our research and for taking the time to review thoroughly. We have tried to make the necessary amendments and additional improvements to the research accordingly. The following are the line-by-line response for your comments | | | |
| No | **Comments** | | **Response** |
| 1. | Was a data transformation used in the meta-analysis? As specified below, the values in several plots did not appear to be based on prevalence directly. Maybe a transformation was applied? | | Yes, we have used log transformation for this meta-analysis. Because prevalence values can vary widely across studies, that can make it distribution of data skewed to extreme values. We used log transformation to normalize the distribution by compressing variability between studies. This allows us to use standard statistical techniques that assume normal distribution and to have more accurate estimate by reducing the impact of extreme values. |
| 2. | Table 1. Add a column of prevalence so that all the information is together. | | This comment was well admitted and correction was made accordingly |
| 3. | Table 2. It says log(prevalence) in the column header, but shouldn’t all values be <0 since prevalence cannot be bigger than 1? Why a log-transformation is used? | | We changed the column header to make it clearer. This table is about the sensitivity analysis to investigate the influence of each individual study on the overall meta-analysis summary estimate. The mataninf command presents a table and a graph of the results of an influence analysis in which the meta-analysis is reestimated by omitting each study at a time in turn. In this analysis the table and the graph (see fig 4) showed consistent result that there was no individual study influence on the overall estimate i.e all “omitted” analysis estimates lied with in the “combined’ analysis confidence interval (last raw).  Each rows of the table represents the meta-analysis estimates of all studies except the "omitted" study named in that row. |
| 4 | Table 3. Please give a more detail description of the sample techniques in “others” group. The same comment applies to the “Others” group for Study Design. | | This comment was well admitted and correction was made accordingly |
| 5 | Figure 3. What is ”b” in the plot? A transformation of the prevalence? | | This is a very valid comment and we clarified on page 13 of the manuscript.  b/se = logprevalence*precision / standard error |
| 6 | Figure 4. Again, what is the estimate? A transformation of the prevalence? | | This is almost the same issue with question number 3 that this is about the graph and the previous are about the tables. Each circled points named “estimate” is the overall summary statistics except the “omitted” study and the horizontal lines represent the CI. The “combined” results are shown as the solid vertical lines with CI from the left and right. |
| 7 | Figure 7. Funnel plot: Was Log(prevalence) shown on the x-axis? Shouldn’t all the values be <0 since prevalence is between 0 and 1. | | Thank you for this important concern. The funnel was plotted with logprevalence on the x-axis against the standard error of logprevalence on the y-axis to assess the potential presence of publication bias. The assumption is that there is no publication bias if all studies lie within the inverted funnel. Small studies scattered to the bottom and large studies cluster to the estimated effect size. |
| 8 | It is worth delving deeper into the causes of heterogeneity. Over half of the studies were in Ethiopia. Does restricting studies to Ethiopia-only alleviate the heterogeneity? Can any factor explain the heterogeneity? | | Thank you for this important comment. As you rightly said, we were worried about the heterogeneity and accumulation of studies in Ethiopia. We tried to investigate the heterogeneity and conduct subgroup analyses with various characteristics, but the heterogeneity persisted. Some literature says that heterogeneity is inhabitable in prevalence studies because of sampling variability and differences in study methods (sampling techniques and data collection tools). The other reasons are the number of included studies (as studies increase, variability increases) and the difference in prevalence (less than 10 and greater than 90 percent can have less heterogeneity). We stated this in the discussion part.  We used the standard search strategy and inclusion and exclusion criteria for studies from LMICs, but we came up with half of the studies being in Ethiopia. As a result, we tried to find studies from other countries by cross-referencing, but the gap didn’t narrow. We discussed this in the discussion and limitation part. |
| For Editor – First of all, I would like to express my appreciation and gratitude for your valuable comments and summary of reviews that significantly improve the quality of our research and for taking the time to review thoroughly. We have tried to make the necessary amendments and additional improvements to the research accordingly. The following are the line-by-line response for your comments | | | |
| No | **Comments** | | **Response** |
| 1. | In your manuscript, you mentioned that 317 articles were selected from PubMed Central based on your search strategy. However, the PRISMA flow chart indicates an initial identification of 8,115 records through PubMed database screening.  Could you kindly elaborate on the primary screening process on PubMed pages? Specifically, why was this step performed before using Rayyan for further selection?  Additionally, how were the 211 possible duplicates identified by the Rayyan system resolved? Were they excluded during the PubMed screening or at a later stage? | | This is a very critical comment, and we made corrections accordingly. We caused confusion for this part while making revisions to the previous comment. This would be difficult if it were published with these errors. Thank you for this major review. |
| 2. | From the PRISMA Flow Chart, you state that PubMed was the sole database used in this systematic review.  However, you also mention retrieving 112 studies from other sources. It remains unclear whether these “other sources” refer to databases such as EMBASE, MEDLINE, Global Health, African Journals Online (AJOL), and Google Scholar.  To enhance transparency, could you explicitly clarify which databases fall under the category of “other sources”? If these databases include EMBASE, MEDLINE, and others, it would be essential to explain why this selection was made. | | This is also a very pertinent comment that we admitted well and corrected accordingly.  We planned to review databases like EMBASE, MEDLINE, Global Health, African Journals Online (AJOL), and Google Scholar in addition to Pubmed as other sources. This was written in the protocol. However, in the actual review, all 112 articles were retrieved from Google Scholar.  When we wrote the result, we overlooked this issue and remained as it was on the protocol. So now we have corrected it as per your important comments.  Our intention to use Google Scholar was to find articles that were not indexed and could not be found in PubMed. |
