# Supplementary material for: Magnitude of standard precautions practices among healthcare workers in health facilities of Low and Middle Income Countries: A systematic review and meta-analysis
Source: PLoS One. 2024 Apr 30;19(4):e0302282. doi: 10.1371/journal.pone.0302282 (PMC11060540; doi:10.1371/journal.pone.0302282)
Supplement: Attachment — Submitted filename: Response to Reviewer and editor.docx [file pone.0302282.s005.docx]

**Authors’ line-by-line response for reviewers**

| **Title** | | **Magnitude of Standard Precautions Practices among Healthcare Workers in Health Facilities of Low and Middle Income Countries: A Systematic Review and Meta-analysis.** | |
| --- | --- | --- | --- |
| **Name of authors** | | Mengistu Yilma, Girma Taye, and Workeabeba Abebe | |
| For Reviewer 3 – Thank you very much for your critical review of the manuscript that helps us to make significant improvement on the manuscript. The comments addressed major issues on this study. | | | |
| No | **Comments** | | **Response** |
| 1. | Is log transformation used throughout the paper including the forest plot, only that the values in the forest plot were back transformed? This needs to be clearly stated in the paper.  I guess my understanding of prevalence is a proportion while percentage (100* proportion) was used throughout the paper. This is fine. However, the standard errors of log Prevalence in Figure 7 seem to be high. Please double check. Please add standard errors of the prevalence in Table 1 as well. If log transformation was used please added the standard errors of the logPrevalence too. | | As you rightly said, there was an error when we wrote the syntax for selogprevalence that caused problems with the result. So considering your comment, we conducted a re-analysis using Stata 18. We used a point-and-click option in Stata 18 rather than writing syntax. We also use proportion without log transformation rather than percentage. There was a significant change in the results on the funnel plot, and some results that we showed with track changes were in a separate document. We would like to thank you again for your valuable and pertinent comments. |
| For Editor – Thank you very much for your critical concern for the quality of the manuscript to be published. Considering your concern, we did a re-analysis using Stata 18 and resolved the critical issues indicated in the comments. | | | |
| No | **Comments** | | **Response** |
| 1. | **Use of a Single Database:** The current limitations statement acknowledges the use of purposive inclusion criteria and the language of included studies. However, it does not sufficiently address the significant limitation stemming from the utilization of only one database for your literature search. Please expand on this point by discussing the potential impact of this limitation on the comprehensiveness of your review and the generalizability of your findings. | | Actually we used PubMed and Google scholar databases as sources of articles for this study. As indicated in the PRISMA framework checklist, we presented a full electronic search strategy for PubMed. As a rule of thumb, A Measurement Tool to Assess systematic Reviews AMSTRA) checklist recommends to search at least two databases.  As per your comment we discussed the impact of using small number of databases. Separate track change document attached. |
| 2. | **Access to Databases and Language Restrictions:** The brief mention of limited access to databases and the language of included studies warrants further elaboration. It is crucial to explicitly state how these factors might have influenced the selection of studies and the potential biases introduced as a result. | | As per your comment, we briefly mentioned in the limitation part. See the attached track change document.  We appreciate your concern and thank you again. |
