# Supplementary material for: Magnitude of standard precautions practices among healthcare workers in health facilities of Low and Middle Income Countries: A systematic review and meta-analysis
Source: PLoS One. 2024 Apr 30;19(4):e0302282. doi: 10.1371/journal.pone.0302282 (PMC11060540; doi:10.1371/journal.pone.0302282)
Supplement: Attachment — Submitted filename: Response to Reviewer and editor.docx [file pone.0302282.s006.docx]

**Authors’ line-by-line response for reviewers**

| **Title** | | **Magnitude of Standard Precautions Practices among Healthcare Workers in Health Facilities of Low and Middle Income Countries: A Systematic Review and Meta-analysis.** | |
| --- | --- | --- | --- |
| **Name of authors** | | Mengistu Yilma, Girma Taye, and Workeabeba Abebe | |
| For Reviewer 3 – Thank you very much for your critical review of the manuscript that helps us to make significant improvement on the manuscript. The comments addressed major issues on this study. | | | |
| No | **Comments** | | **Response** |
| 1. | The funnel plot can be either plotted as log-odds vs 1/standard error or log-odds vs sample size. Please check the paper by Hunter et al for details. | | Thank you for this valuable comment.  The context in Hunter et al. was different from our proportion meta-analysis. They used log-odds vs sample size for a situation when the underlying proportion is quite extreme (very low or very high). The key findings in their study also explained that **conventional funnel plots** appear to be an inaccurate way of assessing publication bias (PB) in **meta-analyses of proportion studies with extreme proportional outcomes.** We have tried their suggestion, but the result was unreliable and distorted for our study.  Moreover, we reviewed further literatures to decide assessment of publication bias in our case. There are arguments about assessing publication bias in proportion meta-analysis. As publication bias was assumed in the context of comparative studies that studies with positive results are more frequently published than studies with negative results, this doesn’t make sense for proportion studies with no comparison. As a result, studies came up with various suggestions that Barker et al. do not suggest using funnel plots, Egger’s and Begg’s tests for proportional meta-analysis, Wang N. suggests to use these tests but interpreting with caution, and Hunter et al. suggest using study size against log odds to assess publication bias in proportion meta-analysis for proportions with extreme outcomes. After careful consideration of this evidence, we decided to use the funnel plot and Egger’s test at least to visualize the distribution of studies and statistical testing along the pooled result.  We clarified this in the revised manuscript. |
|  | Hunter et al "In meta-analyses of proportion studies, funnel plots were found to be an inaccurate method of assessing publication bias" 2014 Journal of Clinical Epidemiology | | As we explained above Hunter et al. key finding explained “conventional funnel plots appear to be an inaccurate way of assessing publication bias (PB) in meta-analyses of proportion studies with **extreme proportional outcomes.** Thus our proportion was not extreme. |
| For Editor – Thank you very much for your critical concern for the quality of the manuscript to be published. Considering your concern, we did a re-analysis using Stata 18 and resolved the critical issues indicated in the comments. | | | |
| No | **Comments** | | **Response** |
| 1. | It's important to clarify a few points regarding your search to ensure the rigour of your systematic review process:   - Google Scholar as a Search Engine: Google Scholar is a valuable search engine, but it is not a traditional academic database (as you've claimed). It lacks the controlled indexing, sophisticated search features, and quality filters necessary for systematic reviews. Indeed, your PRISMA flowchart currently reflects only a PubMed search. If you used Google Scholar as a database, then the flowchart needs to be updated to accurately depict your search strategy. | | We accepted your explanation about Google scholar and as per the comment, we updated the PRISMA flowchart. We explicitly include Google scholar in the chart. We have attached the PRISMA flow chart as Fig. 1 in the system. |
| 2. | Addressing Limitations: Please expand your limitations section to consider the following:   - What relevant studies might have been missed by not including traditional databases? | | We have accepted these comments and addressed accordingly. We expand the limitations that explained missing relevant studies as selection bias by geographical region, and heterogeneity due to difference in population characteristics in different regions. |
|  | - Could the reliance on PubMed and Google Scholar introduce bias into your study selection? | | Yes, we have also indicated in the limitation part that selection bias, lack of standardization and heterogeneity can be introduced due to different data bases have different criteria to publish articles. |
|  | - How might this impact the generalizability of your findings? | | We explained the above reasons may cause less representativeness, generalizability, and quality of included studies as a result of not including more traditional academic databases in this study. |
